# Supplementary figures and images for: Scrutinising an inscrutable bark-nesting ant: Exploring cryptic diversity in the Rhopalomastix javana (Hymenoptera: Formicidae) complex using DNA barcodes, genome-wide MIG-seq and geometric morphometrics
Source: PeerJ. 2023 Nov 16;11:e16416. doi: 10.7717/peerj.16416 (PMC10657568; doi:10.7717/peerj.16416)

**A**

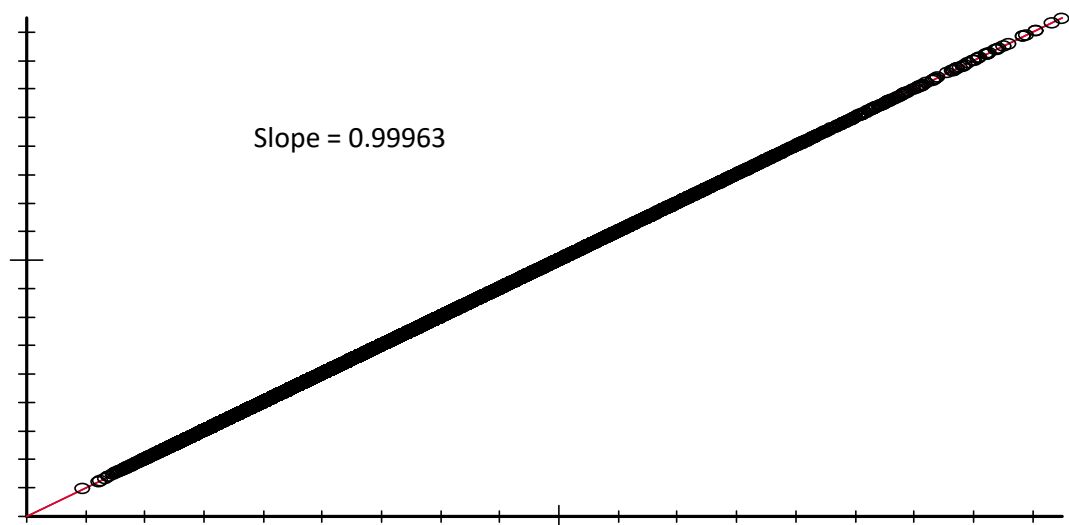

**B**

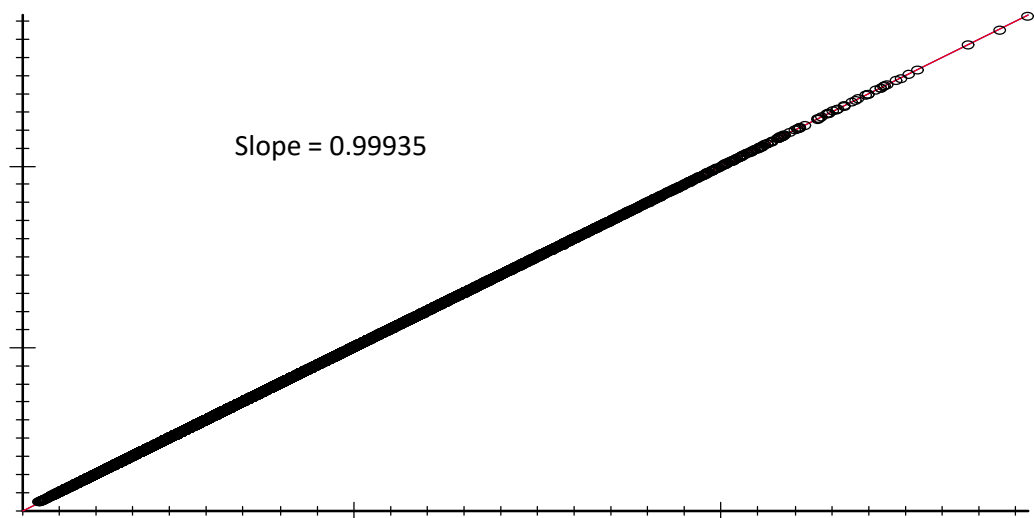

**C**

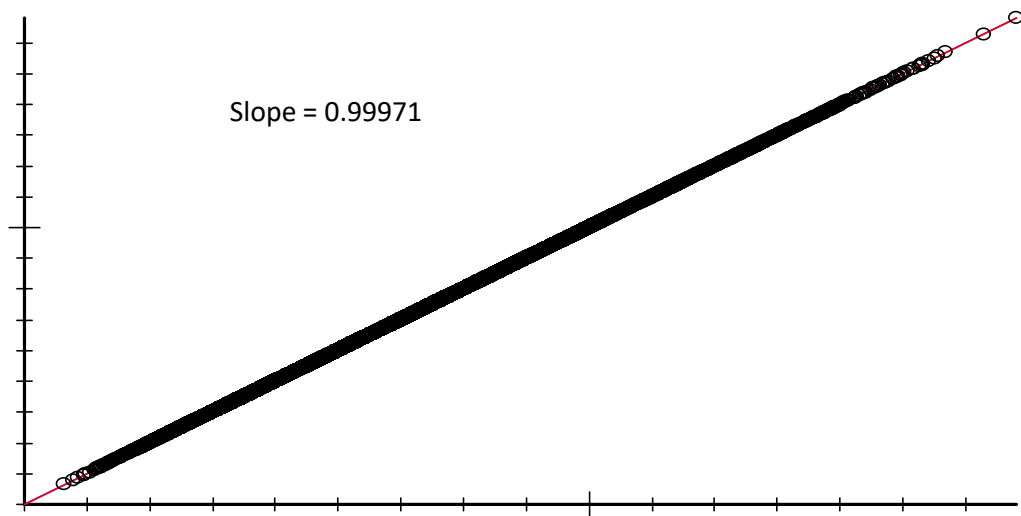

Supplement: Supplemental Information 1 — A: Head, B: Meso, C: Profile. [file peerj-11-16416-s001.pdf]
