# Supplementary material for: Scrutinising an inscrutable bark-nesting ant: Exploring cryptic diversity in the Rhopalomastix javana (Hymenoptera: Formicidae) complex using DNA barcodes, genome-wide MIG-seq and geometric morphometrics
Source: PeerJ. 2023 Nov 16;11:e16416. doi: 10.7717/peerj.16416 (PMC10657568; doi:10.7717/peerj.16416)
Supplement: Supplemental Information 2 — (A) Head, (B) Meso, (C) Profile. [file peerj-11-16416-s002.docx]

| **A. Head - Head in full-face view** | | |
| --- | --- | --- |
| **Point No.** | **Type** | **Description of Position** |
| 1 | Landmark | Posteriormost tip of left eye |
| 2 | Landmark | Anteriormost tip of left eye |
| 3 | Landmark | Posteriormost tip of right eye |
| 4 | Landmark | Anteriormost tip of right eye |
| 5 | Landmark | Supraclypeal area, immediately anterior to anteriormost point of articulation between frontal lobes |
| 6 | Landmark | Median point of anterior clypeal margin |
| 7 | Landmark | Inflection corresponding to lateralmost point of left mandibular insertion |
| 8 | Landmark | Inflection corresponding to lateralmost point of right mandibular insertion |
| 9 | Landmark | Inflection differentiating left-lateral and median portions of clypeus |
| 10 | Landmark | Inflection differentiating right-lateral and median portions of clypeus |
| 11 | Landmark | Anteriormost tip of left frontal lobe |
| 12 | Landmark | Anteriormost tip of right frontal lobe |
| 13 | Landmark | Median depression of posterior head margin |
| 14-23 | Semilandmarks | Lateral margin of left dorsoposterior lobe of head |
| 24-33 | Semilandmarks | Lateral margin of right dorsoposterior lobe of head |
| **B. Meso - Mesosoma in dorsal view** | | |
| **Point No.** | **Type** | **Description of Position** |
| 1 | Landmark | Left inflection corresponding to articulation between propodeum and peduncle |
| 2 | Landmark | Right inflection corresponding to articulation between propodeum and peduncle |
| 3 | Landmark | Left inflection corresponding to articulation between propodeum and mesonotum |
| 4 | Landmark | Right inflection corresponding to articulation between propodeum and mesonotum |
| 5-22 | Semilandmarks | Entire external outline of promesonotal dorsum, including pronotal disc but excluding pronotal neck |
| 23-32 | Semilandmarks | Left lateral propodeal margin |
| 33-42 | Semilandmarks | Right lateral propodeal margin |
| **C. Profile - Mesosoma in profile/lateral view** | | |
| **Point No.** | **Type** | **Description of Position** |
| 1 | Landmark | Dorsalmost point of articulation between peduncle and propodeum |
| 2 | Landmark | Posteriormost articulation point of metacoxa with ventral margin of propodeum |
| 3 | Landmark | Propodeal spiracle orifice |
| 4 | Landmark | Ventralmost point of lateral mesometapleural suture - ventral inflection point between propodeum and mesonotum |
| 5 | Landmark | Ventralmost point of pronotomesopleural suture - ventral inflection point between pronotum and mesonotum |
| 6 | Landmark | Anteriormost articulation point of procoxa with ventral margin of mesosoma (= propleuron) |
| 7 | Landmark | Dorsoanteriormost point of pronotal disc |
| 8-20 | Semilandmarks | Entire dorsal outline of mesosoma from anteriormost point of pronotal disc to distal point of propodeal lobe, including propodeal declivity |
