# Supplementary material for: Scrutinising an inscrutable bark-nesting ant: Exploring cryptic diversity in the Rhopalomastix javana (Hymenoptera: Formicidae) complex using DNA barcodes, genome-wide MIG-seq and geometric morphometrics
Source: PeerJ. 2023 Nov 16;11:e16416. doi: 10.7717/peerj.16416 (PMC10657568; doi:10.7717/peerj.16416)
Supplement: Supplemental Information 3 [file peerj-11-16416-s003.docx]

| **HEAD** |  |  |  |  |  |  |  |  |
| --- | --- | --- | --- | --- | --- | --- | --- | --- |
|  | **Df** | **SS** | **MS** | **Rsq** | **F** | **Z** | **Pr(>F)** |  |
| **species** | 7 | 0.088183 | 0.012598 | 0.289 | 15.6633 | 11.7549 | 0.001 | ** |
| **site** | 5 | 0.018828 | 0.003766 | 0.0617 | 4.6819 | 6.0172 | 0.001 | ** |
| **rep** | 1 | 0.000273 | 0.000273 | 0.00089 | 0.339 | -1.9889 | 0.976 |  |
| **Residuals** | 246 | 0.197851 | 0.000804 | 0.64841 |  |  |  |  |
| **Total** | 259 | 0.305134 |  |  |  |  |  |  |
|  |  |  |  |  |  |  |  |  |
| **MESO** |  |  |  |  |  |  |  |  |
|  | **Df** | **SS** | **MS** | **Rsq** | **F** | **Z** | **Pr(>F)** |  |
| **species** | 7 | 0.08581 | 0.012259 | 0.21047 | 10.2988 | 11.6047 | 0.001 | ** |
| **site** | 5 | 0.02579 | 0.005158 | 0.06325 | 4.3328 | 7.149 | 0.001 | ** |
| **rep** | 1 | 0.00092 | 0.00092 | 0.00226 | 0.7726 | -0.3271 | 0.632 |  |
| **Residuals** | 248 | 0.29521 | 0.00119 | 0.72403 |  |  |  |  |
| **Total** | 261 | 0.40773 |  |  |  |  |  |  |
|  |  |  |  |  |  |  |  |  |
| **PROFILE** |  |  |  |  |  |  |  |  |
|  | **Df** | **SS** | **MS** | **Rsq** | **F** | **Z** | **Pr(>F)** |  |
| **species** | 7 | 0.04728 | 0.006755 | 0.10647 | 4.6778 | 6.3932 | 0.001 | ** |
| **site** | 5 | 0.03824 | 0.007649 | 0.08611 | 5.297 | 6.8949 | 0.001 | ** |
| **rep** | 1 | 0.00047 | 0.000472 | 0.00106 | 0.3271 | -1.8922 | 0.971 |  |
| **Residuals** | 248 | 0.35811 | 0.001444 | 0.80635 |  |  |  |  |
| **Total** | 261 | 0.44411 |  |  |  |  |  |  |
|  |  |  |  |  |  |  |  |  |
| Significance codes: 0 ‘***’ 0.001 ‘**’ 0.01 ‘*’ 0.05 ‘.’ 0.1 ‘ ’ 1 | | | | | |  |  |  |
