# Supplementary material for: Scrutinising an inscrutable bark-nesting ant: Exploring cryptic diversity in the Rhopalomastix javana (Hymenoptera: Formicidae) complex using DNA barcodes, genome-wide MIG-seq and geometric morphometrics
Source: PeerJ. 2023 Nov 16;11:e16416. doi: 10.7717/peerj.16416 (PMC10657568; doi:10.7717/peerj.16416)
Supplement: Supplemental Information 4 — 80.77% of individuals correctly classified to their pre-defined groups/species. [file peerj-11-16416-s004.docx]

| **Species** | **jsp1** | **jsp2** | **jsp3** | **jsp4** | **jsp5** | **out_murgrp** | **murphyi** | **glabri** | **Actual Total** |
| --- | --- | --- | --- | --- | --- | --- | --- | --- | --- |
| **jsp1** | 6 | 0 | 0 | 0 | 2 | 0 | 0 | 0 | 8 |
| **jsp2** | 0 | 1 | 0 | 0 | 5 | 0 | 0 | 0 | 6 |
| **jsp3** | 0 | 0 | 37 | 5 | 4 | 0 | 0 | 0 | 46 |
| **jsp4** | 0 | 0 | 4 | 31 | 5 | 0 | 0 | 0 | 40 |
| **jsp5** | 2 | 5 | 11 | 4 | 110 | 2 | 0 | 0 | 134 |
| **out_murgrp** | 0 | 0 | 0 | 0 | 0 | 9 | 1 | 0 | 10 |
| **murphyi** | 0 | 0 | 0 | 0 | 0 | 0 | 10 | 0 | 10 |
| **glabri** | 0 | 0 | 0 | 0 | 0 | 0 | 0 | 6 | 6 |
| **Predicted Total** | 8 | 6 | 52 | 40 | 126 | 11 | 11 | 6 | 260 |
