# Supplementary material for: Scrutinising an inscrutable bark-nesting ant: Exploring cryptic diversity in the Rhopalomastix javana (Hymenoptera: Formicidae) complex using DNA barcodes, genome-wide MIG-seq and geometric morphometrics
Source: PeerJ. 2023 Nov 16;11:e16416. doi: 10.7717/peerj.16416 (PMC10657568; doi:10.7717/peerj.16416)
Supplement: Supplemental Information 8 — Statistically insignificant p-values, i.e., ≥0.05 are highlighted in grey. [file peerj-11-16416-s008.docx]

| **HEAD** | | | | | | | | |
| --- | --- | --- | --- | --- | --- | --- | --- | --- |
| **Species** | **jsp1** | **jsp2** | **jsp3** | **jsp4** | **jsp5** | **out_murgrp** | **murphyi** | **glabri** |
| **jsp1** |  |  |  |  |  |  |  |  |
| **jsp2** | 0.0168 |  |  |  |  |  |  |  |
| **jsp3** | 0.0028 | 0.0028 |  |  |  |  |  |  |
| **jsp4** | 0.0056 | 0.0028 | 0.0028 |  |  |  |  |  |
| **jsp5** | 0.6048 | 0.0056 | 0.0028 | 0.0028 |  |  |  |  |
| **out_murgrp** | 0.0028 | 0.0056 | 0.0028 | 0.0028 | 0.0028 |  |  |  |
| **murphyi** | 0.0028 | 0.0028 | 0.0028 | 0.0028 | 0.0028 | 0.0028 |  |  |
| **glabri** | 0.014 | 0.0616 | 0.0028 | 0.0028 | 0.0028 | 0.0028 | 0.4452 |  |
| **MESO** | | | | | | | | |
| **Species** | **jsp1** | **jsp2** | **jsp3** | **jsp4** | **jsp5** | **out_murgrp** | **murphyi** | **glabri** |
| **jsp1** |  |  |  |  |  |  |  |  |
| **jsp2** | 0.2464 |  |  |  |  |  |  |  |
| **jsp3** | 0.196 | 1 |  |  |  |  |  |  |
| **jsp4** | 0.0056 | 1 | 0.0056 |  |  |  |  |  |
| **jsp5** | 0.0028 | 0.1876 | 0.0028 | 0.0028 |  |  |  |  |
| **out_murgrp** | 0.0028 | 0.0112 | 0.0028 | 0.0028 | 0.0028 |  |  |  |
| **murphyi** | 0.0812 | 0.6104 | 0.1988 | 0.0056 | 0.0028 | 0.0028 |  |  |
| **glabri** | 0.336 | 0.2352 | 1 | 0.0028 | 0.0056 | 0.0084 | 0.5404 |  |
| **PROFILE** | | | | | | | | |
| **Species** | **jsp1** | **jsp2** | **jsp3** | **jsp4** | **jsp5** | **out_murgrp** | **murphyi** | **glabri** |
| **jsp1** |  |  |  |  |  |  |  |  |
| **jsp2** | 0.672 |  |  |  |  |  |  |  |
| **jsp3** | 0.0112 | 0.1764 |  |  |  |  |  |  |
| **jsp4** | 0.0084 | 0.1288 | 0.0028 |  |  |  |  |  |
| **jsp5** | 0.0196 | 0.042 | 0.0084 | 0.126 |  |  |  |  |
| **out_murgrp** | 0.0028 | 0.4928 | 0.028 | 0.0028 | 0.0028 |  |  |  |
| **murphyi** | 0.0112 | 0.0168 | 0.0028 | 0.0028 | 0.0028 | 0.0028 |  |  |
| **glabri** | 1 | 0.1456 | 0.0084 | 0.0812 | 0.1092 | 0.0084 | 0.3696 |  |
