# Supplementary material for: Scrutinising an inscrutable bark-nesting ant: Exploring cryptic diversity in the Rhopalomastix javana (Hymenoptera: Formicidae) complex using DNA barcodes, genome-wide MIG-seq and geometric morphometrics
Source: PeerJ. 2023 Nov 16;11:e16416. doi: 10.7717/peerj.16416 (PMC10657568; doi:10.7717/peerj.16416)
Supplement: Supplemental Information 9 — Statistically insignificant p-values, i.e., ≥0.05 are highlighted in grey. [file peerj-11-16416-s009.docx]

| A. HEAD |  |  |  |  |
| --- | --- | --- | --- | --- |
| Site | STH | WTH | CTH | SIN |
| STH |  |  |  |  |
| WTH | 0.4392 |  |  |  |
| CTH | 0.0006 | 0.0102 |  |  |
| SIN | 0.0006 | 0.0006 | 0.0006 |  |
| B. MESO | |  |  |  |
| Site | STH | WTH | CTH | SIN |
| STH |  |  |  |  |
| WTH | 0.0024 |  |  |  |
| CTH | 0.0006 | 0.0012 |  |  |
| SIN | 0.0798 | 0.0018 | 0.0006 |  |
| C. PROFILE | |  |  |  |
| Site | STH | WTH | CTH | SIN |
| STH |  |  |  |  |
| WTH | 0.045 |  |  |  |
| CTH | 0.0018 | 0.0012 |  |  |
| SIN | 0.0174 | 0.0018 | 0.003 |  |
