# Supplementary material for: Scrutinising an inscrutable bark-nesting ant: Exploring cryptic diversity in the Rhopalomastix javana (Hymenoptera: Formicidae) complex using DNA barcodes, genome-wide MIG-seq and geometric morphometrics
Source: PeerJ. 2023 Nov 16;11:e16416. doi: 10.7717/peerj.16416 (PMC10657568; doi:10.7717/peerj.16416)
Supplement: Supplemental Information 10 [file peerj-11-16416-s010.docx]

| **s/n** | **Specimen_ID** | **GenBank Accession No.** |
| --- | --- | --- |
| 1 | WW27-28-A_murphyi_SIN | OR262214 |
| 2 | WW27-28-B_murphyi_SIN | OR262215 |
| 3 | WW27-28-C_murphyi_SIN | OR262216 |
| 4 | WW27-28-D_murphyi_SIN | OR262217 |
| 5 | WW20-21-A_jsp5_SIN | OR262218 |
| 6 | WW20-21-B_jsp5_SIN | OR262219 |
| 7 | WW20-21-C_jsp5_SIN | OR262220 |
| 8 | WW20-21-D_jsp5_SIN | OR262221 |
| 9 | WW20-21-E_jsp5_SIN | OR262222 |
| 10 | WW20-21-F_jsp5_SIN | OR262223 |
| 11 | WW20-21-G_jsp5_SIN | OR262224 |
| 12 | WW20-21-H_jsp5_SIN | OR262225 |
| 13 | WW01-H_jsp5_SIN | OR262226 |
| 14 | WW01-C_jsp5_SIN | OR262227 |
| 15 | WW01-A_jsp5_SIN | OR262228 |
| 16 | WW01-F_jsp5_SIN | OR262229 |
| 17 | WW01-I_jsp5_SIN | OR262230 |
| 18 | WW01-E_jsp5_SIN | OR262231 |
| 19 | WW01-B_jsp5_SIN | OR262232 |
| 20 | WW01-D_jsp5_SIN | OR262233 |
| 21 | WW01-G_jsp5_SIN | OR262234 |
| 22 | WW16-17-C_jsp5_STH | OR262235 |
| 23 | WW16-17-D_jsp5_STH | OR262236 |
| 24 | WW16-17-A_jsp5_STH | OR262237 |
| 25 | WW33-E_jsp5_STH | OR262238 |
| 26 | WW33-A_jsp5_STH | OR262239 |
| 27 | WW33-C_jsp5_STH | OR262240 |
| 28 | WW33-D_jsp5_STH | OR262241 |
| 29 | WW33_jsp5_STH | OR262242 |
| 30 | WW30-31-D_jsp5_STH | OR262243 |
| 31 | WW30-31-E_jsp5_STH | OR262244 |
| 32 | WW30-31-A_jsp5_STH | OR262245 |
| 33 | WW30-31-B_jsp5_STH | OR262246 |
| 34 | WW30-31-C_jsp5_STH | OR262247 |
| 35 | WW30-31-F_jsp5_STH | OR262248 |
| 36 | WW32-A_jsp5_STH | OR262249 |
| 37 | WW34-A_jsp5_STH | OR262250 |
| 38 | WW34-B_jsp5_STH | OR262251 |
| 39 | WW32-C_jsp5_STH | OR262252 |
| 40 | WW32-B_jsp5_STH | OR262253 |
| 41 | WW34_jsp5_STH | OR262254 |
| 42 | WW34-C_jsp5_STH | OR262255 |
| 43 | WW32-D_jsp5_STH | OR262256 |
| 44 | WW36-D_jsp5_STH | OR262257 |
| 45 | WW36-B_jsp5_STH | OR262258 |
| 46 | WW36-A_jsp5_STH | OR262259 |
| 47 | WW36-C_jsp5_STH | OR262260 |
| 48 | WW35_jsp5_WTH | OR262261 |
| 49 | WW42-43-A_jsp5_CTH | OR262262 |
| 50 | WW38-39-B_jsp5_CTH | OR262263 |
| 51 | WW49-50-A_jsp5_WTH | OR262264 |
| 52 | WW49-50-B_jsp5_WTH | OR262265 |
| 53 | WW49-50-C_jsp5_WTH | OR262266 |
| 54 | WW40-41-B_jsp5_CTH | OR262267 |
| 55 | WW37-C_jsp5_CTH | OR262268 |
| 56 | WW10-A_jsp3_NTH | OR262269 |
| 57 | WW10-B_jsp3_NTH | OR262270 |
| 58 | WW10-C_jsp3_NTH | OR262271 |
| 59 | WW10-D_jsp3_NTH | OR262272 |
| 60 | WW10-E_jsp3_NTH | OR262273 |
| 61 | WW10-F_jsp3_NTH | OR262274 |
| 62 | WW10-G_jsp3_NTH | OR262275 |
| 63 | WW10-H_jsp3_NTH | OR262276 |
| 64 | WW02-B_jsp3_NTH | OR262277 |
| 65 | WW02-D_jsp3_NTH | OR262278 |
| 66 | WW02-A_jsp3_NTH | OR262279 |
| 67 | WW03-A_jsp3_CTH | OR262280 |
| 68 | WW03-D_jsp3_CTH | OR262281 |
| 69 | WW03-B_jsp3_CTH | OR262282 |
| 70 | WW03-C_jsp3_CTH | OR262283 |
| 71 | WW47-48-C_jsp3_CTH | OR262284 |
| 72 | WW22-23-A_jsp3_CTH | OR262285 |
| 73 | WW22-23-D_jsp3_CTH | OR262286 |
| 74 | WW22-23-B_jsp3_CTH | OR262287 |
| 75 | WW22-23-C_jsp3_CTH | OR262288 |
| 76 | WW05-B_jsp4_NTH | OR262289 |
| 77 | WW05-D_jsp4_NTH | OR262290 |
| 78 | WW05-F_jsp4_NTH | OR262291 |
| 79 | WW05-G_jsp4_NTH | OR262292 |
| 80 | WW05-H_jsp4_NTH | OR262293 |
| 81 | WW06-07-B_jsp4_NTH | OR262294 |
| 82 | WW06-07-C_jsp4_NTH | OR262295 |
| 83 | WW06-07-D_jsp4_NTH | OR262296 |
| 84 | WW08-C_jsp4_NTH | OR262297 |
| 85 | WW08-D_jsp4_NTH | OR262298 |
| 86 | WW08-F_jsp4_NTH | OR262299 |
| 87 | WW08-H_jsp4_NTH | OR262300 |
| 88 | WW04-B_jsp4_NETH | OR262301 |
| 89 | WW04-D_jsp4_NETH | OR262302 |
| 90 | WW04-A_jsp4_NETH | OR262303 |
| 91 | WW04-E_jsp4_NETH | OR262304 |
| 92 | WW04-C_jsp4_NETH | OR262305 |
| 93 | WW44-A_jsp2_CTH | OR262306 |
| 94 | WW26-B_jsp1_CTH | OR262307 |
| 95 | WW26-A_jsp1_CTH | OR262308 |
| 96 | WW29-A_glabri_SIN | OR262309 |
| 97 | WW29-B_glabri_SIN | OR262310 |
| 98 | WW29-C_glabri_SIN | OR262311 |
| 99 | WW29-D_glabri_SIN | OR262312 |
| 100 | WW29-E_glabri_SIN | OR262313 |
| 101 | WW29-F_glabri_SIN | OR262314 |
| 102 | WW29-G_glabri_SIN | OR262315 |
| 103 | WW29-H_glabri_SIN | OR262316 |
| 104 | WW25-E_unk_outgrp_MAL | OR262317 |
| 105 | WW25-G_unk_outgrp_MAL | OR262318 |
| 106 | WW25-H_unk_outgrp_MAL | OR262319 |
| 107 | WW25-B_unk_outgrp_MAL | OR262320 |
| 108 | WW25-C_unk_outgrp_MAL | OR262321 |
